# A pilot study of the immunogenicity of a 9-peptide breast cancer vaccine plus poly-ICLC in early stage breast cancer

**DOI:** 10.1186/s40425-017-0295-5

**Published:** 2017-11-21

**Authors:** Patrick M. Dillon, Gina R. Petroni, Mark E. Smolkin, David R. Brenin, Kimberly A. Chianese-Bullock, Kelly T. Smith, Walter C. Olson, Ibrahim S. Fanous, Carmel J. Nail, Christiana M. Brenin, Emily H. Hall, Craig L. Slingluff

**Affiliations:** University of Virginia, Charlottesville, VA 22908 USA

**Keywords:** Breast cancer, immunotherapy, cancer vaccine, cytotoxic T-cell lymphocyte response, peptide, poly-ICLC, TLR3, agonist

## Abstract

**Background:**

Breast cancer remains a leading cause of cancer death worldwide. There is evidence that immunotherapy may play a role in the eradication of residual disease. Peptide vaccines for immunotherapy are capable of durable immune memory, but vaccines alone have shown sparse clinical activity against breast cancer to date. Toll-like receptor (TLR) agonists and helper peptides are excellent adjuvants for vaccine immunotherapy and they are examined in this human clinical trial.

**Methods:**

A vaccine consisting of 9 MHC class I-restricted breast cancer-associated peptides (from MAGE-A1, −A3, and -A10, CEA, NY-ESO-1, and HER2 proteins) was combined with a TLR3 agonist, poly-ICLC, along with a helper peptide derived from tetanus toxoid. The vaccine was administered on days 1, 8, 15, 36, 57, 78. CD8^+^ T cell responses to the vaccine were assessed by both direct and stimulated interferon gamma ELIspot assays.

**Results:**

Twelve patients with breast cancer were treated: five had estrogen receptor positive disease and five were HER2 amplified. There were no dose-limiting toxicities. Toxicities were limited to Grade 1 and Grade 2 and included mild injection site reactions and flu-like symptoms, which occurred in most patients. The most common toxicities were injection site reaction/induration and fatigue, which were experienced by 100% and 92% of participants, respectively. In the stimulated ELIspot assays, peptide-specific CD8^+^ T cell responses were detected in 4 of 11 evaluable patients. Two patients had borderline immune responses to the vaccine. The two peptides derived from CEA were immunogenic. No difference in immune response was evident between patients receiving endocrine therapy and those not receiving endocrine therapy during the vaccine series.

**Conclusions:**

Peptide vaccine administered in the adjuvant breast cancer setting was safe and feasible. The TLR3 adjuvant, poly-ICLC, plus helper peptide mixture provided modest immune stimulation. Further optimization is required for this multi-peptide vaccine/adjuvant combination.

**Trial registration:**

ClinicalTrials.gov (posted 2/15/2012): NCT01532960. Registered 2/8/2012. https://clinicaltrials.gov/show/NCT01532960

**Electronic supplementary material:**

The online version of this article (10.1186/s40425-017-0295-5) contains supplementary material, which is available to authorized users.

## Background

Immunotherapy for the treatment of cancer is a rapidly expanding field encompassing monoclonal antibodies, bispecific antibodies, T-cell engineering, numerous types of vaccines and an ever growing list of immune stimulating agents. Many of the contemporary immunotherapies in development have the same ultimate goal of inducing anti-cancer responses in an otherwise immunosuppressed tumor microenvironment. Breast cancers utilize several mechanisms to render the tumor environment unfavorable to the effects of the human immune system. Recently, clinical trials of several immunotherapy agents have broken immune tolerance in patients with triple negative breast cancer, offering new promise for other immune therapies [1, 2].

A current challenge in immunotherapy for breast cancer is how to break immune tolerance, which is especially challenging in estrogen receptor positive disease types. One approach is the use of multi-peptide vaccines, which have the potential to expand T lymphocytes against tumor antigens. Peptide-based vaccines administered with appropriate adjuvants can induce antigen-specific T-cell responses against cancer-related antigens [3–7]. A key component of an effective vaccine is a functional adjuvant to enhance the ability of dendritic cells (DC) to generate specific immune responses. In murine models, CD40 agonists have all of these qualities [8–10]; however, CD40 agonists are currently not available for clinical use. As an alternative, we utilize a tetanus helper peptide known to stimulate CD4^+^ T cells [11, 12]. Stimulated CD4+ T cells express CD40L, which in turn should bind CD40 on DC.

Pathogen recognition receptors, such as the TLRs, are useful secondary adjuvant agents in peptide vaccines. TLRs constitute a receptor family that recognizes a wide variety of conserved microbial molecular patterns. TLR3 recognizes double stranded viral RNA, and when the TLR3 receptor on a DC is bound, the DC is rapidly activated to produce cytokines and upregulate co-stimulatory receptors, resulting in vigorous T cell responses [13]. There are data for the use of TLR agonists by topical, intradermal, subcutaneous, intramuscular and systemic routes [13–16], which prompts the current investigation into use of poly-ICLC (a TLR3 agonist) by intradermal and intramuscular routes.

To test the immunogenicity of this novel adjuvant system, we designed a clinical trial combining poly-ICLC with the tetanus helper peptide [11, 12]. Based on prior clinic experience with a 9 peptide vaccine in breast cancer, the poly-ICLC and tetanus peptide were given in conjunction with a 9 peptide mixture of breast cancer associated antigens. The peptides represent portions of the MAGE-A1, −A3, −A10, CEA, NY-ESO-1, and HER2 proteins. One CEA peptide is modified at one amino acid for major histocompatibility complex (MHC) binding affinity [17, 18]. Several of the peptides have already demonstrated excellent immunogenicity in human patients, and one peptide (HER2_369–377_) is in phase III trials as a stand-alone peptide vaccine [19–25]. The peptides for this study (Table 1) were previously employed in other cancer vaccine trials in an emulsion of Incomplete Freund’s Adjuvant (IFA), and they were found to be safe in combination [26]. However, there has been concern from the Overwijk group at MD Anderson that administration of vaccines with IFA might result in antigen-specific T-cells homing back to the vaccine site rather than tumor sites [27–29]. Thus, the adjuvant system described here was developed without IFA.

**Table 1:** Breast cancer related peptides employed in the 9 peptide vaccine and the tetanus peptide adjuvant

Allele	Sequence	Epitope
HLA-A1	EADPTGHSY	MAGE-A1 _161–169_
	EVDPIGHLY	MAGE-A3 _168–176_
HLA-A2	KIFGSLAFL	Her-2/neu _369–377_
	YLSGADLNL	CEA _571–579_*
	GLYDGMEHL	MAGE-A10 _254–262_
HLA-A3	HLFGYSWYK	CEA _27–35_
	VLRENTSPK	Her-2/neu _754–762_
	SLFRAVITK	MAGE-A1 _96–104_
HLA-A3/HLA-A31	ASGPGGGAPR	NY-ESO-1 _53–62_
Tetanus toxoid-derived helper peptide		
Binds to multiple class II alleles	AQYIKANSKFIGITEL	p2_830–844_**

*Asparagine to aspartic acid change at position 576

**An alanine residue was added to the N-terminus to prevent cyclization

We tested the hypothesis that vaccination with a multi-peptide vaccine combined with poly-ICLC and a helper peptide would be safe in the adjuvant breast cancer setting. We also hypothesized that the replacement of IFA by Poly-ICLC as an adjuvant would increase the proportion of participants with ELIspot positive responses to the vaccine. A sub-aim was to assess whether the concurrent use of endocrine therapy had an impact on immunogenicity of the vaccine.

## Methods

This study was a single-arm, open label, pilot study of safety and immune efficacy of peptide vaccination with poly-ICLC and it was open to enrollment by patients with stage IB-IV resected breast cancer (Table 2). In the end, the trial actually enrolled a cohort of patients with stage II and III disease. Participants must have completed their last dose/treatment of any radiation, chemotherapy or trastuzumab therapy between 45 and 180 days prior to enrollment. The study was approved by the University of Virginia Institutional Review Board on 11/2/2011 (IRB#15881). All participants signed approved informed consent per institutional standards. The study was conducted in accordance with declaration of Helsinki with good clinical practice as defined by the International Conference on Harmonization.

**Table 2:** Patient Characteristics

Characteristics	Safety set	Immunologic set
	*n* = 12	*n* = 11
Age in years, median (range)	48 (31, 62)	49 (31, 62)
	n (%)	n (%)
Gender		
Female	12 (100%)	11 (100%)
Race		
Caucasian	12 (100%)	11 (100%)
Ethnicity		
Non-Hispanic	12 (100%)	11 (100%)
Menopausal Status		
Post-menopausal	8 (67%)	7 (64%)
Pre-menopausal	4 (33%)	4 (33%)
Pathologic Type		
Ductal	11 (92%)	11 (100%)
Unknown	1 (8%)	
Histologic Grade		
Grade II	3 (25%)	3 (27%)
Grade III	8 (67%)	7 (64%)
Unknown	1 (8%)	1 (9%)
Stage		
II	3 (25%)	3 (27%)
III	9 (75%)	8 (73%)
ER Status		
Negative	5 (42%)	5 (45%)
Positive	7 (58%)	6 (55%)
PR Status		
Negative	6 (50%)	6 (55%)
Positive	6 (50%)	5 (45%)
Her 2/Neu Status		
Negative	6 (50%)	5 (45%)
Positive	5 (42%)	5 (45%)
Unknown	1 (8%)	1 (9%)
On Hormonal Therapy		
No	6 (50%)	5 (45%)
Yes	6 (50%)	6 (55%)
Type		
Arimidex	3	3
Tamoxifen	3	3
HLA type (may be multiple)		
A1	7 (58%)	7 (64%)
A2	5 (42%)	4 (36%)
A3	6 (50%)	6 (55%)

For each vaccination, the participants received 100 mcg of each of the 9 peptides (Table 1), plus 200 mcg of the tetanus toxoid peptide [12], plus 1 mg poly-ICLC. [30–32] The nine epitopes in the vaccine were chosen based on 1) their MHC restriction (approximately 60–80% of the breast cancer patient population express HLA-A1, −A2, −A3, or -A31), 2) the frequency of expression of the parent protein in adenocarcinomas of the breast, and 3) their proven immunogenicity in vivo*.*

### Dosing and vaccine preparation

Peptides were vialed under sterile condition per FDA approval (BB-IND# 12761). The poly-ICLC (Hiltonol; Oncovir, Inc.; Washington, DC) was provided by the Ludwig Institute (New York, NY) as a clinical grade reagent for experimental use in single-use vials containing 1 mL of a 2 mg/mL solution (IND 43984).

Peptides for vaccines were synthesized and purified (> 95%) under GMP (good manufacturing practice) conditions (Multiple Peptide Systems, now Polypeptide Laboratories, San Diego, CA). The peptides were reconstituted and vialed in single-use vials by either Merck Biosciences AG Clinalfa (Läufelingen, Switzerland) (tetanus peptide) or by the University of Virginia Human Immune Therapy Center (9-peptide vaccine). Each vaccine was 1 ml of a stable solution consisting of 100 mcg of each of the 9 Class I MHC restricted peptides, 200 mcg of the tetanus helper peptide and 1 mg of Poly-ICLC.

Vaccines were administered on days 1, 8, 15, 36, 57, and 78. The vaccine was administered intramuscular (IM) (0.5 ml) and intradermal (ID) (0.5 ml) at one site, alternating between the arm site opposite the breast cancer and an anterior thigh site. All participants were closely observed for adverse events for at least 20 min following each vaccination.

### Participant selection

Eligibility requirements included age ≥ 18 years, ECOG performance 0–1, and expression of HLA-A1, −A2, or -A3. All races and ethnic backgrounds were eligible. Staging was determined using the Seventh Edition AJCC staging system.

Participants were permitted to receive hormonal therapy at the time of study if hormonal therapy was a component of the standard of care. Exclusion criteria included the presence of autoimmune disease, HIV, Hepatitis C, poorly controlled diabetes, pregnancy, known or suspected allergies to vaccine components, or impaired hepatic or renal function.

### Clinical assessments

The study was conducted on an outpatient basis in the University of Virginia Cancer Center with evaluations on days 1, 8, 15, 36, 57, 78, 85 and 108. Participants were off treatment follow-up approximately 4 months after first vaccine administration.

Participants kept a daily diary of toxicities for days 1 through 85. The diaries were reviewed by a research clinician prior to the next scheduled vaccine. The trial was monitored continuously for treatment-related adverse events, using NCI Common Terminology Criteria for Adverse Events version 4.03. Dose-modification of vaccine was indicated if grade 3 adverse events occurred. Protocol treatment was to be discontinued for any grade 3 toxicity.

### Laboratory assessments

**Fig. 1:**
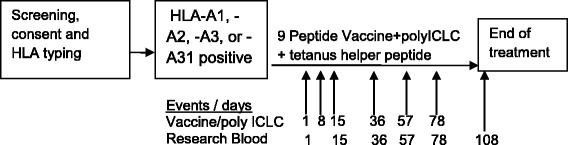
Schema. The general schema of treatment including the timing of the vaccine series and the times for blood draws for ELIspot analysis is shown

PBMC and serum were isolated from peripheral blood (100–140 ml) at the time points shown in fig. 1. Lymphocytes were isolated using Ficoll gradient centrifugation, and cryopreserved in 10% DMSO/90% serum by the Biorepository and tissue Research Facility at the University of Virginia. The ELIspot assay was used to evaluate CD8^+^ T cell responses [33–36]. In this assay, antigen-specific CD8^+^ T cell responses are quantified by IFNγ production. Cytotoxic T-cells that are not anergized should secrete IFNγ after exposure to their cognate antigen, especially if they have a memory phenotype. ELIspot assays can reproducibly detect functional CTL responses to defined antigens at levels below 0.01%. The IFN-γ production is based on the fact that the assay was performed after stimulation with peptides known to be restricted by Class I MHC. Responses to tetanus peptide from CD4^+^ cells were measured similarly.

The ELIspot assays were performed directly ex vivo, after cryopreservation (direct ELIspot) or after one in vitro sensitization (stimulated ELIspot). Methods for the stimulated ELIspot assay have been reported [35, 37]. For direct ELIspot assays, 200,000 peripheral blood mononuclear cells (PBMC) were plated per well, and pulsed with synthetic peptide (10 mcg/ml), in quadruplicate. Controls included irrelevant peptides, a mixture of viral peptides (CEF peptide pool), PMA-ionomycin and PHA. Assessment of immunologic response was based upon a fold-increase over the maximum of two negative controls and the following criteria. Evaluation of T-cell responses was based on the definitions: N_vax_ = number T-cells responding to vaccine peptide; N_neg_ = number T-cells responding to maximum negative control; R_vax_ = N_vax_/N_neg_. A patient was considered to have a T-cell response to vaccination (binary yes/no), by direct ELIspot assay only if all of the following criteria were met: (1) N_vax_ exceeded N_neg_ by at least 20 cells / 100,000 CD4^+^ or CD8^+^ cells (0.02%), where CD8 and CD4 counts were based on flow cytometric evaluations of the PBMC samples. (2) R_vax_ ≥ 2, (3) (N_vax_ − 1 SD) ≥ (N_neg_ + 1 SD), and (4) R_vax_ after vaccination ≥2 × R_vax_ pre-vaccine, as described. The same criteria applied for stimulated ELIspot assays except that the threshold for criterion (1) was higher: such that N_vax_ had to exceed N_neg_ by at least 100 cells / 100,000 CD4^+^ or CD8^+^ cells (0.1%). Fold-increases less than one (e.g., control counts exceed number of responding T-cells, or fold response compared to baseline is less than one) were set equal to one to indicate no response and to prevent overinflating adjusted fold-increases due to pre-vaccine ratios less than one, or division by zero, while not affecting the determination of response. These methods are consistent with our prior analyses [38]. For the CEA peptide, both wild type peptide and modified CEA were assessed.

This pilot study was designed to test for an immune response rate by direct ELIspot of 45% (rate observed in prior study) [26] versus an alternative rate of 75% with a one-sided exact test providing >90% power (actual type II error 0.0775) at an alternative positive rate of 75% assuming a one-sided 10% level test (actual type I error 0.0871) with a target accrual goal of 24 eligible participants. With this design the null hypothesis of a 45% immune response rate would be rejected if 12 or more responses by direct ELIspot were observed in 24 patients.

## Results

Fifteen breast cancer patients consented to screen for the study, and 13 patients were enrolled. One enrolled patient was unable to receive study treatment because of scheduling delays after a prior surgery. An unplanned interim look of the data occurred after accrual of half the target number of participants, thus, a total of 12 patients were included in the safety analysis. One patient experienced disease progression after 2 doses of vaccine and withdrew from the study. Eleven patients completed all vaccines and had research blood drawn at all designated time-points (Fig. 1). The study was terminated early due to the lack of observed responses by direct ELIspot assay in the first 11 participants. Patient demographics and clinical presentations were typical for a cohort of patients with mixed stages of breast cancer (Table 2). The median age was 48. All patients were female, and all were Caucasian. One-half of patients (6 of 12) were on adjuvant tamoxifen or aromatase inhibitor, and those six patients remained on hormonal therapy during the vaccine series.

Treatment-related adverse events are detailed in Table 3. The vaccines were very well tolerated and there were no related late toxicities beyond 30 days post-vaccination. There were no grade 3 or 4 adverse events or treatment-related deaths. There were no dose-limiting toxicities. The most common adverse event was an injection site reaction, which was observed in 100% of participants. Vaccine sites in the leg and arm were generally erythematous and firm, but no ulceration was observed. Nine of the patients had grade 2 injection site reactions, 3 were grade 1. Interestingly the injection site reactions did not persist as long in this study as in prior breast and melanoma vaccine studies (most were less than a week in duration). Less common events included fatigue, fever, chills, nausea, arthralgia, myalgia and headache. Two patients described flu-like symptoms.

**Table 3:** Toxicities and highest grade

BREAST 41 Toxicities (Related)		N = 12	
		Total	
Category	AE	G1	G2
EAR AND LABYRINTH DISORDERS	TINNITUS	1	
GASTROINTESTINAL DISORDERS	NAUSEA	3	
GENERAL DISORDERS AND ADMINISTRATION SITE CONDITIONS	CHILLS	3	
	FATIGUE	6	5
	FEVER	4	
	FLU LIKE SYMPTOMS	2	
	INJECTION SITE REACTION	3	9
IMMUNE SYSTEM DISORDERS	AUTOIMMUNE DISORDER	2	
INJURY, POISONING AND PROCEDURAL COMPLICATIONS	BRUISING	1	
	SEROMA	1	
METABOLISM AND NUTRITION DISORDERS	ANOREXIA	3	
MUSCULOSKELETAL AND CONNECTIVE TISSUE DISORDERS	ARTHRALGIA	6	
	MYALGIA	5	
	OTHER	1	
NERVOUS SYSTEM DISORDERS	DIZZINESS	3	
	HEADACHE	5	1
PSYCHIATRIC DISORDERS	AGITATION	1	
	OTHER	2	
SKIN AND SUBCUTANEOUS TISSUE DISORDERS	HYPERHIDROSIS	1	
VASCULAR DISORDERS	FLUSHING	1	
OVERALL MAXIMUM		3	9

**Fig. 2:**
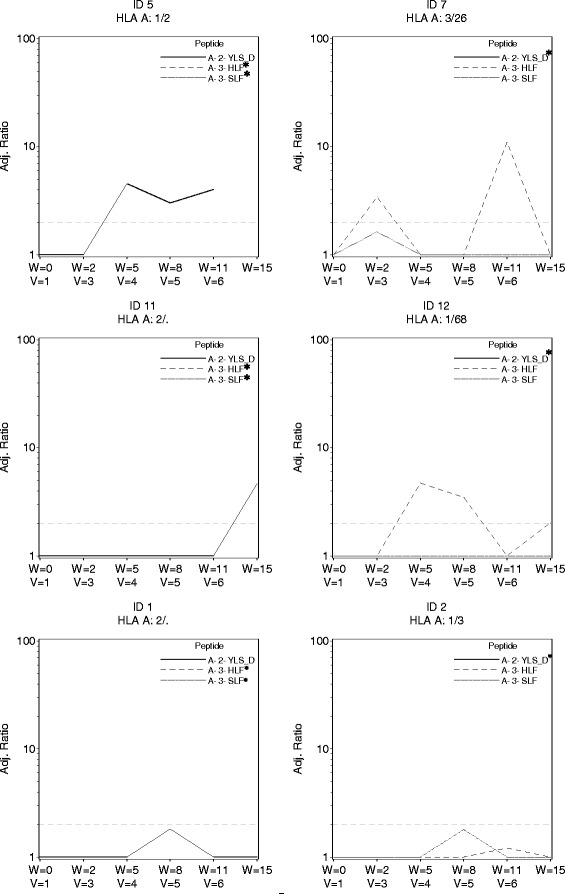
Stimulated ELIspot responses. Four confirmed responses and 2 near-responders to the multi-peptide vaccine following in vitro stimulated and analyzed by ELIspot. The x-axis shows both the week of study and the vaccine number (v = 1, etc). The y axis is label for the adjusted ratio of spots to negative control. The dashed line indicates the preferred threshold ratio for response and is set at a threshold ratio of 2.0 and minimum of 20 T cells per 100,000 CD8+ T cells in a stimulated assay. Only the HLA relevant peptides for each patient are shown. *In all graphs there is at least one peptide (marked *) for which the adjusted ELIspot ratio remained 0 throughout and the corresponding data points for ratio of 0 are not shown

The trial was designed with use of a direct ex vivo ELIspot assay as the primary assay for evaluating CD8^+^ T cell responses to peptides in the vaccine. No responses were observed to the 9 breast peptides in the direct ex vivo ELIspot assays in the first eleven participants [90% CI(0, 23.4%)] which indicated an immune response rate below the null rate of 45%. Nine patients had responses to tetanus helper peptide. As part of a secondary endpoint for the study, we used the stimulated ELIspot assay. Four of the eleven evaluable participants had CD8+ T cell responses with that assay. Two additional patients had responses just below the cut-off for positivity. The four immune responders and two near responders are displayed, over time, in Fig. 2. Notably patients 5, 7 and 12 had positive ELIspot assays at multiple time points, suggesting persistent immune recognition. Three of the four responders had their first ELIspot responses detectable by week 5. Two ELISpot responses were observed in response to the modified HLA-A2 CEA_571–579_ peptide (YLS-D antigen) and two responses were observed in response to HLA-A3 CEA_27–35_ (HLF antigen). One patient had a borderline response to HLA-A3 MAGE-A1_96–104_ (patient 2). Surprisingly neither of the HER2 peptides in this study generated strong ELIspot response.

## Discussion

Peptide vaccines are appealing both for prevention and for treatment purposes of cancer. Their promise is that they may activate and expand T cells capable of immediate tumor rejection and may generate T-cell memory to provide long-term protection from tumor recurrence. The challenges facing peptide vaccines in breast cancer relate to overcoming the low immunogenicity of estrogen receptor positive cancers, the locally immunosuppressive microenvironment, poor DC activation and perhaps tumor heterogeneity/plasticity [39]. This trial sought to improve vaccine activity in breast cancer by the addition of a TLR3 agonist and the limitation to patients without high tumor burdens. We included a helper peptide as a mechanism for activating helper T cells [40–45]. Responses to the tetanus helper peptide were observed by ELIspot as expected (Fig. 2 and Additional file 1: Figure S1).

The concept of the vaccine site as a depot, which may attract activated T cells to home back to the vaccine site rather than the tumor sites is a recently studied challenge to cancer vaccines [27–29]. The current trial was designed to avoid both the T cell depot effects of IFA and also to avoid the toxicity of using IFA as an adjuvant (i.e. vaccine site ulceration/infection/pain in >30% of patients) [46–48]. For these reasons IFA was excluded from the preparation of this 9 peptide vaccine. In the absence of IFA, the toxicity of this vaccine was minimal; there were markedly low rates of local reactions and no injection site ulceration was observed. GM-CSF was considered for use an adjuvant, but given a prior report showing negative impact on immunogenicity [49] it was omitted.

The results from the stimulated ELIspot assays in this trial indicate that circulating T cell responses to vaccinating peptides may be observed. The observation of response to the CEA peptide is encouraging since CEA is somewhat aberrantly expressed by many breast cancers, both ER positive and negative [50, 51]. This observation that T cells could be stimulated to recognize CEA corroborates several prior CEA-based vaccines [52–55], most notably a CEA DNA vaccine resulted in clonal, CEA-specific T cell responses as well as B-cell responses and helper responses [56].

Furthermore, the observation of ELIspot response to MAGE-A1 in breast cancer is intriguing since there are recently reports of immunogenicity of MAGE-A1 for breast cancer patients [57–60]. It was previously reported that 27% of breast tumors are positive for at least one of the MAGE transcripts and MAGE expression was more common in ductal breast cancer and Ki-67 high tumors [61]. Thus, our observation of MAGE-A1 response in a small sample of ductal breast cancer patients and observation in both ER positive and ER negative patients adds support to future investigation of this peptide for cancer vaccine research.

Notably the HER2_369–377_ peptide is the same peptide which was reported to generate robust immune responses in several prior trials when combined with GM-CSF [19–25]. We did not observe strong responses to the HER2 peptides, despite the fact that these peptides are reported to generate T cell responses in other studies [62]. The sample size of HLA A2/A3 patients for HER2 peptides is too small to draw a conclusion, but it is probable that the adjuvant system employed in our study was inadequate. It might also be a possibility that with nearly half of the patients in this study showing baseline overexpression of HER2, tolerance toward the HER2 protein could pre-date vaccination. Indeed HER2 immunity has been reported to be lost in clinically HER2 positive disease and that may also explain our observations [63].

Adjuvants have been employed in vaccines for over 80 years with varying degrees of benefit across many disease types. Many TLR agonists have shown success as adjuvants. Poly-ICLC is one TLR3 agonist which is often combined with vaccine and cellular immunotherapies in order to induce type I interferons and mimic inflammatory response to systemic viral infection by amplification of interferons alpha and gamma as well as IL-1a and IL-6 [15, 30, 32, 64]. The addition of poly-ICLC to immunotherapies generally seems to augment the breadth and strength of the CD8 T cell response and in some cases generates anti-tumor activity [15, 30, 32, 64]. Poly-ICLC trials have acceptable safety profiles, even when poly-ICLC has been given systemically [15, 32, 64–68]. Despite these compelling data, we were unable to determine whether the administration of poly-ICLC by intra-dermal and intramuscular routes had an impact on vaccine efficacy due to the low observed immune response rates in this study.

Overall, the addition of a poly-ICLC and tetanus helper to a multipeptide vaccine proved to be safe and well tolerated in both estrogen receptor positive patients and triple negative patients. As a pilot study, several encouraging immune responses were observed by stimulated ELIspot assays in both estrogen receptor positive and estrogen receptor negative participants (notably this trial was designed prior to an understanding of triple negative breast cancer as a comparatively more immunogenic subtype). Unfortunately, immune responses to the direct ELIspot assay were below the levels observed for a similar 9-peptide vaccine trial in breast cancer [26] and were below the target threshold for progression to a phase II study with this particular combination and administration schedule. This pilot study was closed early with a final sample size of 12 patients due to the absence of immune response in direct ELIspot assays and perceived futility of reaching the primary endpoint for immune response. The response rates and final sample size did not permit robust assessment of endocrine therapy, although responses were observed in both endocrine-treated patients and non-treated patients. No adverse interaction between endocrine therapy and vaccine therapy was observed.

This pilot study is one of several recent or ongoing vaccine studies administered in the post-surgical adjuvant setting. Our peptide vaccine appears to be tolerable in this adjuvant setting, in agreement with other vaccine studies in the adjuvant setting (as opposed to the metastatic setting) [69]. Several peptides were immunogenic in this breast cancer population.

Future directions for this research will include the use of novel phosphopeptide antigens, the use of daily poly-ICLC, the addition of a CD40 antibody, the addition of alternative TLR agonists and vaccination with larger pools of helper peptides and analysis of Th1 and Th2 responses (IL-10, IL-4, IL-5, etc) to a more robust helper peptide approach. Furthermore, given the growing interest in tumor microenvironment, it will be useful to assess whether peptide vaccines impact on the macrophage, DC and T cell infiltration and activation in and around the local tumor sites. Results of tumor biopsy studies and pre-operative vaccine studies will help guide future study designs. Another direction may be the use of nanoparticles for direct delivery of antigens or RNA to DC’s [70].

## Conclusion

This pilot study of immunogenicity of a TLR3 agonist in combination with helper T cell stimulation as adjuvants to traditional class I peptides showed that novel adjuvants can be safely combined with multi-peptide vaccines in the high risk breast cancer setting. Furthermore, the specific peptides to CEA and MAGE-A1 were shown to be immunogenic in this population. The administration of vaccines in estrogen receptor positive patients was shown to be feasible and safe. Some of the estrogen receptor positive patients were able to generate T cells response to peptide vaccines. The design of future breast cancer peptide studies should optimize the use of TLR agonists, IFA, nanoparticles and helper peptides and should further examine the tumor microenvironment in molding the response or lack of response to peptide vaccines.

## Additional file

Additional file 1: Supplemental Data. (DOCX 68 kb)

## Abbreviations

BB-IND: Biologic based investigational new drug
DC: dendritic cell
GMP: Good manufacturing practice
ID: Intradermal
IFA: Incomplete Freund’s Adjuvant
IM: Intramuscular
MHC: Major histocompatibility complex
PBMC: peripheral blood mononuclear cells
TLR: Toll like receptor
TLR3: Toll like receptor 3
