# Supplementary material for: A pilot study of the immunogenicity of a 9-peptide breast cancer vaccine plus poly-ICLC in early stage breast cancer
Source: J Immunother Cancer. 2017 Nov 21;5:92. doi: 10.1186/s40425-017-0295-5 (PMC5697108; doi:10.1186/s40425-017-0295-5)
Supplement: Additional file 1: — Supplemental Data. (DOCX 68 kb) [file 40425_2017_295_MOESM1_ESM.docx]

**Additional file 1**

**Table S1:** Full response data for participants with a response by stimulated assay.

| ID | Reg ID | VBC | HLA-A1 | HLA-A2 | Assay Type | Time Point | Type | HLA | Peptide | Ratio- Adj | Diff- Adj |
| --- | --- | --- | --- | --- | --- | --- | --- | --- | --- | --- | --- |
| 5 | 11951 | 79 | 1 | 2 | Stimulated | W=5/V=4 | A | 1 | EVD | 1.00 | 0.00 |
| 5 | 11951 | 79 | 1 | 2 | Stimulated | W=5/V=4 | A | 2 | GLY | 1.00 | 0.00 |
| 5 | 11951 | 79 | 1 | 2 | Stimulated | W=5/V=4 | A | 2 | KIF | 1.00 | 0.00 |
| 5 | 11951 | 79 | 1 | 2 | Stimulated | W=5/V=4 | A | 2 | YLS_D | 4.55 | 1928.46 |
| 5 | 11951 | 79 | 1 | 2 | Stimulated | W=5/V=4 | A | 2 | YLS_N | 1.00 | 0.00 |
| 5 | 11951 | 79 | 1 | 2 | Stimulated | W=8/V=5 | A | 1 | EAD | 1.00 | 0.00 |
| 5 | 11951 | 79 | 1 | 2 | Stimulated | W=8/V=5 | A | 1 | EVD | 1.00 | 0.00 |
| 5 | 11951 | 79 | 1 | 2 | Stimulated | W=8/V=5 | A | 2 | GLY | 1.00 | 0.00 |
| 5 | 11951 | 79 | 1 | 2 | Stimulated | W=8/V=5 | A | 2 | KIF | 1.00 | 0.00 |
| 5 | 11951 | 79 | 1 | 2 | Stimulated | W=8/V=5 | A | 2 | YLS_D | 3.01 | 955.23 |
| 5 | 11951 | 79 | 1 | 2 | Stimulated | W=8/V=5 | A | 2 | YLS_N | 1.00 | 0.00 |
| 5 | 11951 | 79 | 1 | 2 | Stimulated | W=11/V=6 | A | 1 | EAD | 1.00 | 0.00 |
| 5 | 11951 | 79 | 1 | 2 | Stimulated | W=11/V=6 | A | 1 | EVD | 1.00 | 0.00 |
| 5 | 11951 | 79 | 1 | 2 | Stimulated | W=11/V=6 | A | 2 | GLY | 1.00 | 0.00 |
| 5 | 11951 | 79 | 1 | 2 | Stimulated | W=11/V=6 | A | 2 | KIF | 1.00 | 0.00 |
| 5 | 11951 | 79 | 1 | 2 | Stimulated | W=11/V=6 | A | 2 | YLS_D | 4.03 | 1631.65 |
| 5 | 11951 | 79 | 1 | 2 | Stimulated | W=11/V=6 | A | 2 | YLS_N | 1.00 | 0.00 |
| 7 | 11985 | 81 | 3 | 26 | Stimulated | W=2/V=3 | A | 3 | ASG | 1.00 | 0.00 |
| 7 | 11985 | 81 | 3 | 26 | Stimulated | W=2/V=3 | A | 3 | HLF | 3.42 | 822.20 |
| 7 | 11985 | 81 | 3 | 26 | Stimulated | W=2/V=3 | A | 3 | SLF | 1.62 | 136.63 |
| 7 | 11985 | 81 | 3 | 26 | Stimulated | W=2/V=3 | A | 3 | VLR | 1.00 | 0.00 |
| 7 | 11985 | 81 | 3 | 26 | Stimulated | W=5/V=4 | A | 3 | ASG | 1.00 | 0.00 |
| 7 | 11985 | 81 | 3 | 26 | Stimulated | W=5/V=4 | A | 3 | HLF | 1.00 | 0.00 |
| 7 | 11985 | 81 | 3 | 26 | Stimulated | W=5/V=4 | A | 3 | SLF | 1.00 | 0.00 |
| 7 | 11985 | 81 | 3 | 26 | Stimulated | W=5/V=4 | A | 3 | VLR | 1.00 | 0.00 |
| 7 | 11985 | 81 | 3 | 26 | Stimulated | W=8/V=5 | A | 3 | ASG | 1.00 | 0.00 |
| 7 | 11985 | 81 | 3 | 26 | Stimulated | W=8/V=5 | A | 3 | HLF | 1.00 | 0.00 |
| 7 | 11985 | 81 | 3 | 26 | Stimulated | W=8/V=5 | A | 3 | SLF | 1.00 | 0.00 |
| 7 | 11985 | 81 | 3 | 26 | Stimulated | W=8/V=5 | A | 3 | VLR | 1.00 | 0.00 |
| 7 | 11985 | 81 | 3 | 26 | Stimulated | W=11/V=6 | A | 3 | ASG | 1.00 | 0.00 |
| 7 | 11985 | 81 | 3 | 26 | Stimulated | W=11/V=6 | A | 3 | HLF | 10.99 | 3525.26 |
| 7 | 11985 | 81 | 3 | 26 | Stimulated | W=11/V=6 | A | 3 | SLF | 1.00 | 0.00 |
| 7 | 11985 | 81 | 3 | 26 | Stimulated | W=11/V=6 | A | 3 | VLR | 1.00 | 0.00 |
| 7 | 11985 | 81 | 3 | 26 | Stimulated | W=15 | A | 3 | ASG | 1.00 | 0.00 |
| 7 | 11985 | 81 | 3 | 26 | Stimulated | W=15 | A | 3 | HLF | 1.00 | 0.00 |
| 7 | 11985 | 81 | 3 | 26 | Stimulated | W=15 | A | 3 | SLF | 1.00 | 0.00 |
| 7 | 11985 | 81 | 3 | 26 | Stimulated | W=15 | A | 3 | VLR | 1.00 | 0.00 |
| 11 | 12024 | 85 | 2 | . | Stimulated | W=15 | A | 2 | GLY | 1.00 | 0.00 |
| 11 | 12024 | 85 | 2 | . | Stimulated | W=15 | A | 2 | KIF | 1.00 | 0.00 |
| 11 | 12024 | 85 | 2 | . | Stimulated | W=15 | A | 2 | YLS_D | 4.63 | 277.51 |
| 11 | 12024 | 85 | 2 | . | Stimulated | W=15 | A | 2 | YLS_N | 1.00 | 0.00 |
| 12 | 12030 | 86 | 1 | 68 | Stimulated | W=5/V=4 | A | 1 | EAD | 1.00 | 0.00 |
| 12 | 12030 | 86 | 1 | 68 | Stimulated | W=5/V=4 | A | 1 | EVD | 1.00 | 0.00 |
| 12 | 12030 | 86 | 1 | 68 | Stimulated | W=5/V=4 | A | 3 | ASG | 1.00 | 0.00 |
| 12 | 12030 | 86 | 1 | 68 | Stimulated | W=5/V=4 | A | 3 | HLF | 4.72 | 1422.59 |
| 12 | 12030 | 86 | 1 | 68 | Stimulated | W=5/V=4 | A | 3 | SLF | 1.00 | 0.00 |
| 12 | 12030 | 86 | 1 | 68 | Stimulated | W=5/V=4 | A | 3 | VLR | 1.00 | 0.00 |
| 12 | 12030 | 86 | 1 | 68 | Stimulated | W=8/V=5 | A | 1 | EAD | 1.00 | 0.00 |
| 12 | 12030 | 86 | 1 | 68 | Stimulated | W=8/V=5 | A | 1 | EVD | 1.00 | 0.00 |
| 12 | 12030 | 86 | 1 | 68 | Stimulated | W=8/V=5 | A | 3 | ASG | 1.00 | 0.00 |
| 12 | 12030 | 86 | 1 | 68 | Stimulated | W=8/V=5 | A | 3 | HLF | 3.47 | 1610.71 |
| 12 | 12030 | 86 | 1 | 68 | Stimulated | W=8/V=5 | A | 3 | SLF | 1.00 | 0.00 |
| 12 | 12030 | 86 | 1 | 68 | Stimulated | W=8/V=5 | A | 3 | VLR | 1.00 | 0.00 |
| 12 | 12030 | 86 | 1 | 68 | Stimulated | W=11/V=6 | A | 1 | EAD | 1.00 | 0.00 |
| 12 | 12030 | 86 | 1 | 68 | Stimulated | W=11/V=6 | A | 1 | EVD | 1.00 | 0.00 |
| 12 | 12030 | 86 | 1 | 68 | Stimulated | W=11/V=6 | A | 3 | ASG | 1.00 | 0.00 |
| 12 | 12030 | 86 | 1 | 68 | Stimulated | W=11/V=6 | A | 3 | HLF | 1.00 | 0.00 |
| 12 | 12030 | 86 | 1 | 68 | Stimulated | W=11/V=6 | A | 3 | SLF | 1.00 | 0.00 |
| 12 | 12030 | 86 | 1 | 68 | Stimulated | W=11/V=6 | A | 3 | VLR | 1.00 | 0.00 |
| 12 | 12030 | 86 | 1 | 68 | Stimulated | W=15 | A | 1 | EAD | 1.00 | 0.00 |
| 12 | 12030 | 86 | 1 | 68 | Stimulated | W=15 | A | 1 | EVD | 1.00 | 0.00 |
| 12 | 12030 | 86 | 1 | 68 | Stimulated | W=15 | A | 3 | ASG | 1.00 | 0.00 |
| 12 | 12030 | 86 | 1 | 68 | Stimulated | W=15 | A | 3 | HLF | 2.03 | 325.63 |
| 12 | 12030 | 86 | 1 | 68 | Stimulated | W=15 | A | 3 | SLF | 1.00 | 0.00 |
| 12 | 12030 | 86 | 1 | 68 | Stimulated | W=15 | A | 3 | VLR | 1.00 | 0.00 |

**Table S2**

| **BREAST 41 Toxicities (Any Relation)** | | N=12 ----- Total | | | | |
| --- | --- | --- | --- | --- | --- | --- |
| Category | AE | G1 | G2 | G3 | G4 | G5 |
| EAR AND LABYRINTH DISORDERS | TINNITUS | 2 |  |  |  |  |
| GASTROINTESTINAL DISORDERS | CONSTIPATION | 1 |  |  |  |  |
|  | MUCOSITIS ORAL | 3 |  |  |  |  |
|  | NAUSEA | 3 |  |  |  |  |
| GENERAL DISORDERS AND ADMINISTRATION SITE CONDITIONS | CHILLS | 4 |  |  |  |  |
|  | EDEMA LIMBS | 1 |  |  |  |  |
|  | FATIGUE | 5 | 6 |  |  |  |
|  | FEVER | 5 |  |  |  |  |
|  | FLU LIKE SYMPTOMS | 2 |  |  |  |  |
|  | INJECTION SITE REACTION | 3 | 9 |  |  |  |
|  | PAIN |  | 2 |  |  |  |
| IMMUNE SYSTEM DISORDERS | AUTOIMMUNE DISORDER | 2 |  |  |  |  |
| INFECTIONS AND INFESTATIONS | PHARYNGITIS | 1 |  |  |  |  |
| INJURY, POISONING AND PROCEDURAL COMPLICATIONS | BRUISING | 2 |  |  |  |  |
|  | SEROMA | 1 |  |  |  |  |
| INVESTIGATIONS | LYMPHOCYTE COUNT DECREASED |  | 2 |  |  |  |
|  | OTHER |  | 1 |  |  |  |
| METABOLISM AND NUTRITION DISORDERS | ANOREXIA | 3 |  |  |  |  |
|  | HYPERGLYCEMIA |  | 1 |  |  |  |
|  | HYPOGLYCEMIA |  | 1 |  |  |  |
| MUSCULOSKELETAL AND CONNECTIVE TISSUE DISORDERS | ARTHRALGIA | 5 | 1 |  |  |  |
|  | BONE PAIN | 1 |  |  |  |  |
|  | MYALGIA | 5 | 1 |  |  |  |
|  | OTHER | 1 |  |  |  |  |
| NERVOUS SYSTEM DISORDERS | DIZZINESS | 5 |  |  |  |  |
|  | HEADACHE | 6 | 1 |  |  |  |
|  | PRESYNCOPE |  | 1 |  |  |  |
| PSYCHIATRIC DISORDERS | AGITATION | 1 |  |  |  |  |
|  | OTHER | 2 |  |  |  |  |
| REPRODUCTIVE SYSTEM AND BREAST DISORDERS | PELVIC PAIN | 1 |  |  |  |  |
| RESPIRATORY, THORACIC AND MEDIASTINAL DISORDERS | ALLERGIC RHINITIS | 2 |  |  |  |  |
|  | COUGH | 1 |  |  |  |  |
|  | DYSPNEA | 1 |  |  |  |  |
|  | NASAL CONGESTION | 2 |  |  |  |  |
|  | SORE THROAT | 2 |  |  |  |  |
| SKIN AND SUBCUTANEOUS TISSUE DISORDERS | HYPERHIDROSIS | 1 |  |  |  |  |
|  | OTHER | 1 |  |  |  |  |
|  | RASH ACNEIFORM | 1 |  |  |  |  |
| SURGICAL AND MEDICAL PROCEDURES | OTHER |  | 1 |  |  |  |
| VASCULAR DISORDERS | FLUSHING | 1 |  |  |  |  |
|  | HOT FLASHES | 1 |  |  |  |  |
| *OVERALL MAXIMUM* | **** |  | 12 |  |  |  |

**Figure S1.** Direct ELIspot count numbers adjusted for background.
